# Nestin as a Vascular Marker of Angiogenesis in Non-Melanoma Skin Cancer

**DOI:** 10.3390/cancers18091495

**Published:** 2026-05-06

**Authors:** Katarzyna Nowogrodzka, Maciej Tota, Aleksandra Piotrowska, Andrzej Bieniek, Piotr Dzięgiel, Alina Jankowska-Konsur

**Affiliations:** 1 University Centre of General Dermatology and Oncodermatology, Wroclaw Medical University, Borowska 213, 50-556 Wroclaw, Poland; 2 Division of Histology and Embryology, Department of Human Morphology and Embryology, Faculty of Medicine, Wroclaw Medical University, 50-368 Wroclaw, Poland

**Keywords:** nestin, intermediate filament, angiogenesis, BCC, basal cell carcinoma, SCC, squamous cell carcinoma, AK, actinic keratosis

## Abstract

Background: Angiogenesis is critical for tumor progression. Microvessel density (MVD) is commonly assessed using CD31 and CD34, which detect both mature and newly formed vessels and therefore cannot distinguish active neoangiogenesis from stable, quiescent vasculature. Nestin, an intermediate filament protein expressed preferentially in proliferating endothelial cells, has been proposed as a complementary marker of active angiogenesis and has been investigated in several solid tumor types, including pancreatic, colorectal, and breast carcinomas. However, no studies have quantitatively compared nestin-positive MVD across AK, BCC, and SCC using standardized methods. Methods: Immunohistochemistry for nestin, CD31, and CD34 was performed on 118 patient samples collected in 2015–2019 and diagnosed with AK, BCC, or SCC. MVD was quantified by averaging vessel counts in three representative “hot spot” areas. Results: Nestin-positive MVD was significantly lower in patients with AK compared to patients with BCC and SCC (*p* < 0.001). The mean MVD of nestin-positive vessels was significantly lower in AK than in BCC and SCC (*p* < 0.0001). In all three groups, nestin-positive MVD demonstrated a strong, positive correlation with both CD34 and CD31. Conclusions: Nestin-positive MVD was significantly elevated in BCC and SCC compared to AK lesions and demonstrated strong correlations with standard angiogenic markers. These findings suggest that nestin may warrant further investigation as a complementary marker of angiogenesis in non-melanoma skin cancer. Whether nestin-positive MVD offers independent diagnostic or prognostic value in this context remains to be determined in larger, prospective, multicentre studies.

## Simple Summary

Widely used vascular markers, such as CD31 and CD34, label both mature and newly formed vascular endothelial cells, which complicates the precise evaluation of active angiogenesis. Nestin, a cytoskeletal protein found in proliferating endothelial cells, has been proposed as a marker of newly formed vascular endothelial cells. In this study, we examined nestin-positive MVD in actinic keratosis (AK), basal cell carcinoma (BCC), and squamous cell carcinoma (SCC) to evaluate its applicability in detecting active angiogenesis. We found that nestin-positive MVD was significantly higher in BCC and SCC than in AK and showed a strong positive correlation with CD31 and CD34. These results suggest that nestin may serve as a complementary marker of angiogenesis in non-melanoma skin cancer. Further studies are needed to determine whether nestin-positive MVD correlates with clinical outcomes such as tumor progression or recurrence.

## 1. Introduction

Actinic keratoses (AK), also referred to as senile keratoses or solar keratoses, are benign intra-epithelial neoplasms. These lesions are irregular, red, scaly papules or plaques on areas of the body that have been exposed to the sun. Prompt identification and the establishment of an appropriate treatment plan are essential, as AK has the potential to progress to invasive squamous cell carcinoma (SCC) [1].

SCC is the second most prevalent skin malignancy following basal cell carcinoma (BCC), and its incidence is rising worldwide. SCC accounts for the majority of metastatic diseases and deaths associated with non-melanoma skin cancer [2]. Risk factors include prolonged UV radiation exposure, immunosuppression, chronic wounds, lower Fitzpatrick skin phototype, male gender, advanced age, genetic syndromes, and a prior history of squamous cell carcinoma [3].

BCC is the most prevalent form of cancer in humans, with around 3.6 million cases diagnosed annually [4]. Major risk factors include prolonged UV radiation exposure, male gender, lower Fitzpatrick skin phototype, advanced age, prolonged immunosuppression, a positive personal or family medical history, and specific genodermatoses. BCC metastasises infrequently, and its associated mortality rate is low; however, it can lead to considerable morbidity. Genetic mutations, particularly within the hedgehog signaling pathway, are significant contributors to the pathogenesis of BCC [5].

Angiogenesis is a complex process involving the formation of new capillaries through the elongation and branching of the pre-existing vascular network, initiated by stimulation from proangiogenic factors. It may occur as part of physiological processes, such as embryogenesis and tissue regeneration, or pathological processes, including carcinogenesis, atherosclerosis, arthritis, psoriasis, endometriosis, obesity, and SARS-CoV-2 infection [6,7]. The process of vascularization is regulated by a dynamic balance between activating and inhibiting factors [8]. The development of a mature vascular network proceeds through successive molecular stages that rely on the coordinated interaction of cells, the extracellular matrix, and angiogenic mediators.

Angiogenic factors—such as increased VEGF levels and enhanced bFGF expression in keratinocytes—appear to play a significant role in the progression of skin cancers [9]. Exposure to UVB radiation alters the balance of angiogenic regulators, promoting endothelial cell proliferation in existing blood vessels via stimulation of bFGF and VEGF, while simultaneously suppressing the production of the antiangiogenic cytokine interferon-β (IFN-β) [10].

The degree of angiogenesis is commonly evaluated using the microvessel density (MVD) index, determined by immunostaining with antibodies against CD34, CD31, and factor VIII [11]. However, a significant limitation of these widely used markers is that they detect endothelial cells in both mature, quiescent vessels and newly formed, actively proliferating vasculature [12,13]. This inability to distinguish between existing vessels and active neoangiogenesis complicates the precise evaluation of current angiogenic activity, which is a key driver of progression and metastasis.

CD34 is a transmembrane glycoprotein belonging to the sialomucin family of adhesion molecules, with a molecular weight of approximately 110 kDa [14]. It is expressed on hematopoietic progenitor cells, mast cells, dendritic cells, endothelial progenitor cells, and mature endothelial cells, and is thus widely used to assess angiogenesis. Immunohistochemical staining for CD34 highlights both large and small vessels of varying maturity.

CD31, also referred to as platelet endothelial cell adhesion molecule-1 (PECAM-1), is a member of the immunoglobulin superfamily. It is expressed on endothelial cells, neutrophils, monocytes, T and B lymphocytes, platelets, and certain tumor cells. CD31 expression occurs in both immature and mature vessels and is also present in lymphatic endothelium. Factor VIII, a glycoprotein essential for blood coagulation, is primarily expressed in large, mature vessels [15]. The MVD index is calculated as the mean number of vessels per mm^2^ in the three most vascularized regions, known as “hot spots” [16]. Another method for evaluating angiogenesis is the Chalkley method, which estimates the area or volume fraction of vessels within the three most vascularized regions [17].

**Figure 1:**
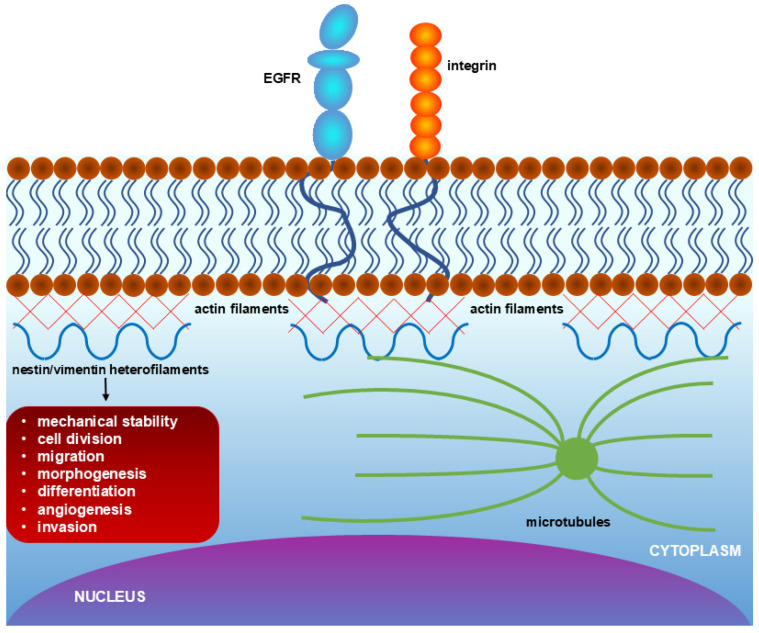
Schematic representation of nestin/vimentin heterofilaments and their cytoskeletal and signaling associations in vascular endothelial cells. The diagram illustrates how nestin/vimentin heterofilaments (blue wavy lines) interact with other cytoskeletal networks—actin filaments (red lines) and microtubules (green lines)—beneath the plasma membrane. Key membrane-associated receptors, such as EGFR (epidermal growth factor receptor) and integrins, transmit extracellular signals into the cytoplasm.

Nestin, a type VI intermediate filament, has emerged as a promising candidate marker of active angiogenesis. Unlike mature markers, nestin expression in adult tissues is largely restricted to areas of cellular proliferation and reorganization [18] [Figure 1]. Nestin interacts with vimentin, desmin, α-internexin, and synemin to form heterodimers [19]. Nestin expression is predominantly observed in proliferating vascular endothelial cells and endothelial progenitor cells. Notably, its expression is more representative of newly formed blood vessels compared to other endothelial cell markers [20]. Nestin plays a crucial role in regulating proliferation, migration, and apoptosis across different cell types [21]. Furthermore, nestin expression has been documented in blood vessels associated with glioblastoma, prostate cancer, colorectal cancer, melanoma, and pancreatic cancer [2,3,4,5,6,7,8,9,10,11,12,13,14,15,16,17,18,19,20,21,22,23,24,25,26,27]. Kleeberger et al. showed that nestin is also implicated in cell migration and metastasis in prostate cancer [25].

Nestin is expressed consistently throughout the early embryonic dermis; however, during later stages of development, its expression is restricted to the connective tissue sheath surrounding hair follicles [28]. During embryogenesis, keratinocytes do not exhibit nestin expression, whereas dendritic cells are nestin-positive, initially dispersed among keratinocytes and later localizing to the outer root sheath. Nestin facilitates the proliferation of neural progenitor cells (NPCs) through the p38-MAPK and EGFR pathways [21]. Developing blood vessels also display nestin positivity throughout embryogenesis [12]. Wang et al. observed nestin expression in the epidermis and the upper two-thirds of the hair follicle in non-balding human scalp skin [29]. Notably, nestin-positive MVD is increased in response to scalp wounds, and the nestin-positive cells appear to derive from the follicular mesenchyme [28].

Nestin is expressed across various tissues and in stem or progenitor cells, including pancreatic islets, skeletal muscle satellite cells, developing myotomes, stem Leydig cells, hair follicles, and the heart, neuroepithelial stem cells, mesenchymal stem cells, and cancer stem cells (CSCs) [30,31,32,33,34,35,36,37]. CSCs are identified as a minor subpopulation of undifferentiated cells within tumor tissue, distinguished by their ability to self-renew and differentiate into diverse lineages and clones that contribute to the formation of tumor masses [38]. CSCs are implicated in cancer initiation, progression, metastasis, therapy resistance, and recurrence [38,39]. One proposed marker for CSCs is nestin, which is frequently co-expressed alongside other stem cell markers, including CD133, Oct3/4, and Sox-2 [38].

The aim of the current study was to evaluate the nestin-positive MVD in vascular endothelial cells within actinic keratosis (AK), basal cell carcinoma (BCC), and squamous cell carcinoma (SCC). Additionally, the study aimed to compare the expression patterns of nestin and microvessel density (MVD) with those identified using conventional angiogenic markers, specifically CD31 and CD34. Furthermore, the investigation sought to ascertain whether nestin could be regarded as a complementary marker of angiogenesis in non-melanoma skin cancer.

## 2. Materials and Methods

### 2.1. Study Population

This retrospective study included 118 patients treated at the Department of Plastic Surgery and the Chair and Clinic of Dermatology, Venereology, and Allergology, Wroclaw Medical University, between 2015 and 2019. Inclusion criteria were as follows:

- Histopathologically confirmed diagnosis of actinic keratosis (AK), basal cell carcinoma (BCC), or squamous cell carcinoma (SCC);
- Availability of archival formalin-fixed, paraffin-embedded (FFPE) tissue blocks with sufficient material for immunohistochemical analysis;
- Complete clinical data available in medical records, including age, sex, lesion location, duration, and relevant clinical history.

Exclusion criteria were as follows:

- Prior treatment of the lesion before biopsy, including cryotherapy, photodynamic therapy, topical therapy, radiotherapy, or surgical intervention;
- Concurrent malignancy other than adequately treated non-melanoma skin cancer at other sites;
- Insufficient tissue sample for completion of the full immunohistochemical panel;
- Poor tissue preservation or extensive processing artifact precluding accurate histological assessment.

The final cohort comprised 47 patients diagnosed with BCC, 39 with AK, and 32 with SCC. Clinical data collected included patient age, sex, lesion location, and lesion duration, defined as the time from the patient’s initial self-detection of the lesion to the date of surgical excision and definitive histopathological diagnosis.

Among patients diagnosed with SCC, 34.4% were female and 65.6% were male. The age range was 55–99 years, with a mean age of 78.6 ± 10.0 years. The mean disease duration was 1.5 ± 1.36 years. The majority of lesions (83.8%) were located on sun-exposed areas of the skin, most commonly on the scalp and nose (each 19.4%). The mean infiltration depth was 4.9 ± 4.2 mm. At the time of diagnosis and surgical excision, none of the 32 patients with SCC presented with clinical or radiological evidence of regional lymph node involvement or distant metastasis, and all cases were therefore classified as primary, non-metastatic cutaneous SCC.

In the BCC group, 53.2% of patients were female and 46.8% were male. The age range was 40–99 years, with a mean age of 71.0 ± 12.9 years. The mean lesion diameter was 15.2 ± 9.0 mm (range: 5–50 mm), and the mean disease duration was 1.8 ± 1.75 years. Multiple lesions were observed in 27.7% of patients, and recurrent lesions were recorded in 8.5% of cases.

Among patients with AK, 71.8% were female and 28.2% were male. The mean age was 70.4 ± 12.5 years, and the mean duration of the condition was 1.4 ± 1.9 years. The demographic, clinical, and histological characteristics of all three groups are summarized in Table 1.

This study was conducted in accordance with the principles of the Declaration of Helsinki and received approval from the Independent Ethics Committee of Wroclaw Medical University (approval no. KB-311/2019).

### 2.2. Immunohistochemistry (IHC)

**Figure 2:**
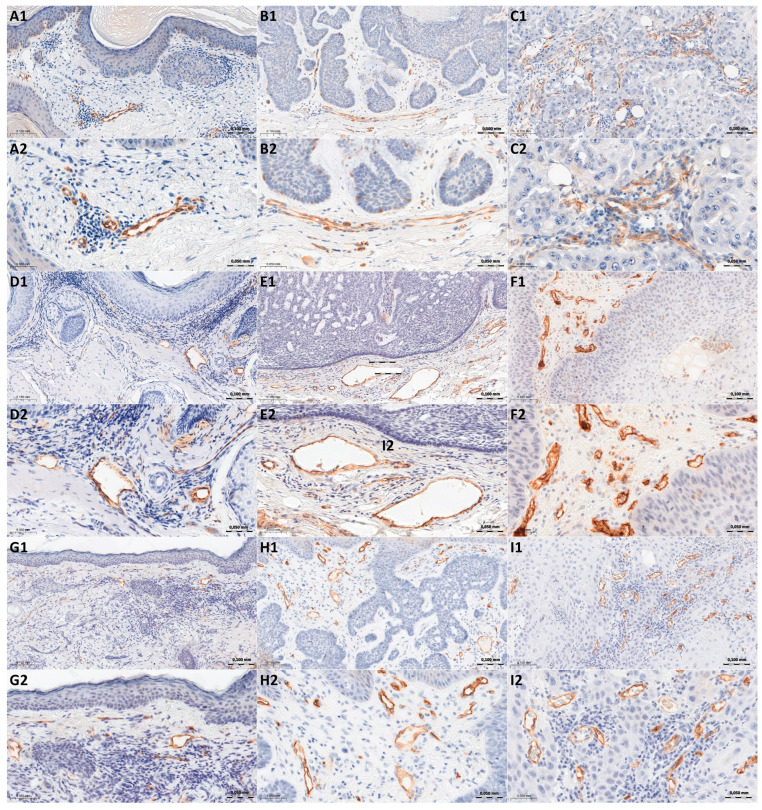
Immunohistochemical expression of Nestin (**A**–**C**) in AK (A), BCC (**B**), and SCC (**C**); CD31 (**D**–**F**) in AK (**D**), BCC (**E**), and SCC (**F**); CD34 (**G**–**I**) in AK (**G**), BCC (**H**), and SCC (**I**). Magnification ×200 (slides marked with number 1), magnification ×400 (slides marked with number 2).

The study was performed on archival paraffin blocks containing 47 cases of basal cell carcinoma and 32 cases of squamous cell carcinoma. A total of 39 cases of actinic keratosis were established as the comparison group. From these materials, 4 μm thick sections were cut and immunohistochemical reactions were performed using primary antibodies: nestin (cat. no. OBT1610, 1:100, BIO-RAD, Hercules, CA, USA), CD31 (cat. no. IR610, ready-to-use, Agilent, Santa Clara, CA, USA), and CD34 (cat. no. IR632, ready-to-use, Agilent). The immunohistochemical reaction procedure using the EnVision FLEX+, Mouse visualization kit (Agilent) included deparaffinization, hydration, and epitope unmasking in Tris/EDTA buffer, pH 9 (EnVision FLEX Target Retrieval Solution, High pH, (for CD31, CD34, and nestin antibodies). Next, in order to block endogenous peroxidase activity, slides were incubated with EnVision FLEX Peroxidase-Blocking Reagent (5 min, RT, Agilent). Subsequently, the sections were incubated with primary antibodies (20 min, RT) and with EnVision FLEX/HRP (20 min, RT) (Agilent). The reactions were visualized using substrate for horseradish peroxidase—DAB (incubation 10 min., RT). Finally, slides were counterstained with FLEX Hematoxylin (Agilent) for 5 min at RT, and dehydrated in graded alcohol concentrations (70%, 96%, 99,8%) and xylene. The slides were mounted in Mounting Medium (Agilent). Figure 2 shows the immunohistochemical presentation of nestin-positive MVD, CD31, and CD34 in AK, BCC, and SCC.

### 2.3. The Microvessel Density (MVD) Assessment

Microvessel density (MVD) was assessed using electronically cataloged virtual slides (Pannoramic MIDI II, 3DHistech Ltd., Sysmex Suisse AG, Horgen, Switzerland) at 200× magnification. Scanned images were viewed and analyzed using Quant Center software version 2.3 (3DHistech). Region of Interest (ROI) areas were standardized using the software’s spatial calibration based on the scanner’s metadata (0.95 mm^2^ per field at 200× magnification). All area measurements were verified against a calibrated digital scale to ensure the accuracy of the vessels/mm^2^ calculation.

According to the literature, CD31 and CD34 are among the most frequently utilized endothelial cell markers for assessing MVD [13,40,41,42,43]. Nestin, which is also expressed in the vascular endothelial cells, was included as an additional marker of blood vessels. The standardized criteria established by Weidner et al. [44] were employed for the counting of microvessels. A countable microvessel was defined as any distinct endothelial cell or cluster of endothelial cells that exhibited positive staining and was clearly distinguishable from adjacent vessels, tumor cells, and connective tissue components. Of note:

- A visible lumen was NOT required for a structure to be counted;
- The presence of red blood cells was NOT required for identification;
- Isolated single positive endothelial cells and small endothelial clusters without a lumen were included, provided they met the separation criteria;
- Branching structures were counted as a single vessel if they were part of the same continuous structure.

The following structures and areas were explicitly excluded from counting:

- Large vessels with thick muscular walls (arteries, arterioles, veins, venules with identifiable smooth muscle layers), as these represent pre-existing mature vasculature rather than tumor-associated neovessels;
- Vessels located within or immediately adjacent to areas of necrosis or ulceration.
- Vessels within dense inflammatory infiltrates where the inflammatory reaction itself could induce non-specific angiogenesis;
- Vessels in areas of sclerosis, section folding, or other artifacts that obscured clear visualization;
- The perichondral and periosteal regions, where present, as these contain normal vascular networks unrelated to the tumor.

To ensure objectivity and minimize bias, all assessments were performed by two independent observers (K.N., and P.D.) who were fully blinded to the clinical diagnosis (AK, BCC, or SCC) and to the antibody marker (nestin, CD31, or CD34). Slides were assigned random codes, and the key was not revealed until after all counts were completed. For each case, the entire slide was reviewed at low magnification (40×) to identify the three areas with the highest vascular density, known as ‘hot spots.’ These hot spots were selected based on the highest concentration of nestin-, CD31-, or CD34-positive vessels.

Once the three hot spots were identified for each marker, vessel counting was performed at 200× magnification. At this magnification, the calibrated field area captured by the scanner corresponds to 0.95 mm^2^ of tissue.

For each of the three hot spots, the total number of positive vessels was recorded. The MVD value for each marker was then calculated as the arithmetic mean of the vessel counts from the three hot spots. All MVD values reported in this study are expressed as the mean number of vessels per square millimeter (vessels/mm^2^).

To ensure consistency, all measurements were performed by two independent observers who were blinded to the clinical diagnosis. In cases of discrepancy, a consensus was reached by simultaneous review.

### 2.4. Statistical Analysis

To address the research questions, statistical analyses were conducted using IBM SPSS Statistics version 28. Basic descriptive statistics were generated, followed by the Shapiro–Wilk test, Student’s *t*-test for independent samples, Pearson’s correlation coefficient, the Mann–Whitney U test and the Kruskal–Wallis test. The normality of the distribution for each variable was verified using the Shapiro–Wilk test prior to the application of Pearson’s correlation coefficient. For normally distributed variables, Pearson’s correlation coefficient was used, with a Bonferroni-adjusted significance level of α = 0.017 to account for multiple comparisons.

Due to the varying sizes of the AK, BCC, and SCC groups, a Kruskal–Wallis analysis was conducted to compare microvessel density (MVD) across the three diagnostic groups for all markers. For group comparisons involving unequal group sizes, the Kruskal–Wallis test with *post hoc* Dunn–Bonferroni correction was employed to identify specific between-group differences. Additionally, pairwise comparisons between individual diagnostic groups were performed using the Mann–Whitney U test, with *p*-values reported both unadjusted and following Bonferroni correction where applicable. For all analyses, statistical significance was set at α = 0.05 unless otherwise specified.

Formal interobserver reproducibility statistics were not prospectively calculated for this cohort. To minimize observer-dependent variability, both observers (K.N. and P.D.) were blinded to all clinical diagnoses, outcomes, and original pathology reports. Slides were de-identified and assigned random codes prior to evaluation to ensure that the assessment of each marker was performed independently of clinical context.

Hotspot selection and vessel counting criteria were standardized according to the methodology of Weidner et al., prior to commencement of the study [44]. In cases where vessel counts between the two observers differed by more than 10% of the higher count within a given hotspot field, a consensus value was established by simultaneous re-review of the relevant field using a dual-headed microscope. Consensus review was required in 15 cases (12.7% of the total cohort), arising most frequently in fields containing dense inflammatory infiltrates or areas of partial necrosis.

## 3. Results

In patients with AK, nestin showed a strong positive correlation with CD34 and a moderate positive correlation with CD31, indicating that increasing nestin-positive microvessel density (nestin-positive MVD) was associated with higher levels of CD31 and CD34. A statistically significant, moderately strong positive correlation was also observed between CD31 and CD34, suggesting coordinated endothelial activation [Table 2]. All *p*-values reported in Table 2, Table 3 and Table 4 represent raw, unadjusted two-tailed *p*-values.

In the BCC cohort, a strong positive correlation was observed between nestin and CD34, and a moderate positive correlation between nestin and CD31. As in AK, a strong correlation was found between CD31 and CD34, demonstrating consistent co-expression of endothelial markers [Table 3].

In the SCC group, nestin was strongly and positively correlated with both CD31 and CD34. CD31 and CD34 were also strongly correlated with each other, indicating synchronous upregulation of endothelial markers in SCC tissue [Table 4].

**Figure 3:**
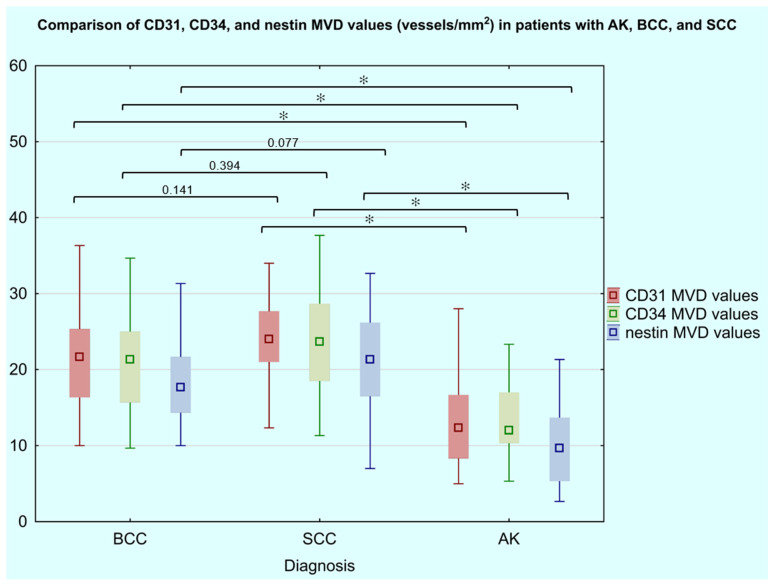
Box plot graphs demonstrating comparative analysis of CD31-, CD34-, and nestin-positive MVD values (vessels/mm^2^) in patients with AK, BCC, and SCC. Numerical *p*-values above the boxplots indicate unadjusted Mann–Whitney U test results for pairwise BCC–SCC comparisons. Asterisks (✻) between the boxplots denote statistically significant differences (*post hoc* Dunn–Bonferroni test, *p* < 0.001).

To determine whether nestin-positive microvessel density (nestin-positive MVD) differed significantly among the three lesion types, a Kruskal–Wallis test was performed [Table 5]. The Kruskal–Wallis test revealed statistically significant differences in nestin-positive MVD across the three diagnostic groups (H(2) = 48.61, *p* < 0.001), with a large overall effect size (η^2^ = 0.405). The mean nestin-positive MVD was 10.15 ± 5.45 vessels/mm^2^ (95% CI: 8.39–11.92) in AK, 18.09 ± 4.73 vessels/mm^2^ (95% CI: 16.70–19.48) in BCC, and 20.99 ± 6.60 vessels/mm^2^ (95% CI: 18.61–23.37) in SCC. *Post hoc* Dunn–Bonferroni comparisons demonstrated that nestin-positive MVD was significantly lower in AK than in both BCC (test statistic = 41.47, SE = 7.39, Z = 5.60, *p* < 0.001, rank-biserial r = 0.745, Cohen’s d = −1.565) and SCC (test statistic = 54.05, SE = 8.13, Z = 6.64, *p* < 0.001, rank-biserial r = 0.804, Cohen’s d = −1.807), with large effect sizes observed for both comparisons [Table 6] [Figure 3]. No statistically significant difference in nestin-positive MVD was identified between BCC and SCC (test statistic = 12.57, SE = 7.77, Z = 1.62, *p* = 0.077, rank-biserial r = 0.295, Cohen’s d = −0.521), although a small-to-moderate effect size was noted. Consistent with these findings, CD31- and CD34-positive MVD followed an identical pattern of group differences, with large effect sizes observed for AK versus malignant group comparisons across all three markers (CD31: rank-biserial r = 0.609 and r = 0.733; CD34: rank-biserial r = 0.668 and r = 0.779, for AK vs. BCC and AK vs. SCC, respectively).

## 4. Discussion

To the best of our knowledge, the current study is among the first to specifically quantify and compare the nestin-positive microvessel density (nestin-positive MVD) in vascular endothelial cells in non-melanoma skin cancer and actinic keratosis. In this study, we investigated nestin expression in the vascular endothelium of AK, BCC, and SCC. The mean density of nestin-positive vessels was significantly higher in BCC and SCC compared to AK, but no significant difference was observed between BCC and SCC. This pattern was consistent with the results obtained via CD31 and CD34 immunostaining. Moreover, strong correlations were observed between nestin and both CD31 and CD34, suggesting that nestin is strongly associated with endothelial activation in these lesions.

The absence of a statistically significant difference in MVD between BCC and SCC—consistently observed across all three vascular endothelial markers—warrants mechanistic consideration. Although BCC and SCC represent biologically and clinically distinct malignancies with divergent growth patterns, invasive behavior, and metastatic potential, both tumor types are well-established to be angiogenesis-dependent, and the present data suggest that their overall vascular requirements, as reflected by MVD, may be broadly comparable at the lesion level assessed in this cohort. Several biological mechanisms may account for this convergence. Both BCC and SCC arise in chronically sun-damaged skin that has undergone prolonged ultraviolet-induced stromal remodeling, including upregulation of vascular endothelial growth factor (VEGF), matrix metalloproteinases, and pro-inflammatory cytokines that collectively create a permissive angiogenic microenvironment prior to frank malignant transformation. Consequently, the vascular compartment within which both tumor types develop may already be substantially activated, potentially limiting the incremental angiogenic stimulus attributable to the tumor itself and thus attenuating detectable between-tumor differences in MVD. Furthermore, the predominant growth pattern of nodular BCC—characterized by expansile, cohesive tumor nests with a well-defined stromal interface—is associated with a pronounced desmoplastic and angiogenic stromal reaction that may sustain MVD at levels comparable to those observed in SCC despite the lower intrinsic metastatic capacity of BCC.

Previous research has demonstrated that in pancreatic cancer, CD34, CD31, and factor VIII are expressed in both newly formed and mature vessels, whereas nestin expression is restricted to newly formed vasculature, supporting its proposed role as a marker of angiogenesis. While nestin gene silencing has been shown to inhibit endothelial cell proliferation in vitro [24,27], suggesting a potential functional role in the angiogenic process, it remains to be determined if nestin exerts similar regulatory effects in cutaneous malignancies. Literature suggests nestin may interact with other cytoskeletal proteins like vimentin to facilitate the shape changes required for new vessel formation [18,19]. Nestin was associated with poor prognosis in other neoplasms [12,28]. Furthermore, the restricted expression pattern of nestin—present in the proliferating endothelium of tumors but absent from quiescent, mature vessels in adult tissues—makes it a putative target. Future research could explore strategies to disrupt the function of nestin or its interaction with partner proteins (like vimentin).

In ductal breast carcinoma, nestin expression has also been linked to angiogenesis and poor prognosis. A study of 124 cases found that nestin expression in tumor cells correlated with nestin-positive endothelial vessels and SOX-18 expression. Elevated nestin-positive MVD was associated with triple-negative breast cancer, high proliferative rates, and worse overall survival. The authors proposed that elevated nestin expression in tumor cells is associated with enhanced angiogenesis; however, the underlying mechanisms remain to be elucidated [45].

Nestin expression as a marker of angiogenesis has also been investigated in colorectal cancer. A positive association was observed between high MVD with nestin-positive vessels and lymph node involvement. Patients with advanced-stage disease exhibited significantly higher MVD indices for nestin-expressing vessels than those with early-stage tumors, suggesting that nestin could be a reliable marker for assessing angiogenesis and tumor progression in colorectal cancer. Notably, nestin identified significantly smaller vessels than CD34, suggesting greater sensitivity for detecting early neovascularization [46].

Collectively, these studies highlight nestin as a reliable marker of proliferating endothelial cells, particularly in neovascularization [47,48,49]. Consequently, it has been suggested in the literature that the application of nestin extends beyond the mere quantification of blood vessels to potentially facilitate a dynamic evaluation of the angiogenic process itself. While our findings show a higher nestin-positive MVD in malignant lesions, the extent to which this allows for a ‘dynamic’ assessment of the angiogenic switch remains a hypothesis that requires further longitudinal investigation.

Angiogenesis, the formation of new blood vessels from pre-existing endothelium, is a critical biological process in tumor growth and metastasis. The extent of tumor angiogenesis is commonly quantified using the microvessel density (MVD) index. Research focused on predicting tumor behavior in non-melanoma skin cancer through the quantification of MVD presents significant challenges. The primary limitation lies in the absence of a universally accepted and optimal vascular marker [50]. Furthermore, considerable variability in staining protocols, antibody clones, and counting methodologies across studies has hampered the comparability of results and the establishment of standardized thresholds. To date, CD31, CD34, BNH9, VEGF, COX-2, HIF1A, factor VIII, VEGFR1, and VEGFR2 have been suggested as the markers of vascular endothelial cells [50,51,52]. However, none of these markers represents an ideal universal standard. Each exhibits distinct advantages and limitations with respect to sensitivity, specificity, and expression patterns across different vascular compartments and tumor microenvironments. For instance, while CD31 and CD34 are among the most widely employed endothelial markers due to their relatively consistent expression in both mature and newly formed vessels, they may also stain lymphatic endothelium and haematopoietic cells, thereby potentially confounding MVD assessments [53,54,55].

Comparative analyses of MVD have revealed significant differences between AK and BCC. Newell et al. demonstrated that MVD, assessed with an anti-CD34 antibody, was significantly higher in both BCC and AK compared to normal skin, with BCC exhibiting a markedly higher density than AK, suggesting a correlation with greater malignancy [56]. Chin et al. further delineated vascular patterns by qualitatively and quantitatively analyzing the microvasculature in BCC, trichoepithelioma, and SCC using anti-CD31. Their findings indicated that invasive growth was associated with a stromal angiogenic response, while metastatic potential correlated with the presence of microvessels within the tumor mass–a feature observed in SCC but not in nodular BCC or trichoepithelioma [57]. Supporting this, Pastuchenko et al. reported a higher vessel count in both the stroma and tumor mass of SCC compared to BCC using CD31 and CD105 markers [58].

Despite these findings, a limitation of conventional markers like CD31 and CD34 is their detection of both nascent and mature vasculature, potentially confounding the specific assessment of angiogenesis [24]. This has prompted the investigation of alternative markers, such as nestin, an intermediate filament protein proposed to be a reliable marker of proliferating endothelial cells during neovascularization. While its role in angiogenesis has been explored in other malignancies, its potential application in skin cancers had not been previously reported.

Notably, the mean density of nestin-positive vessels was slightly lower than the MVD assessed with anti-CD31 and anti-CD34 antibodies. A trend was observed wherein nestin expression was predominantly localized to smaller vessels, while larger vessels remained unstained. This finding aligns with reports from other tumor types. For instance, in gastric adenocarcinoma, MVD labeled with nestin strongly correlated with CD34-based MVD [59]. In colorectal cancer, nestin was identified in endothelial cells of significantly smaller vessels compared to those stained by CD34 [60]. Moreover, in pancreatic cancer, nestin expression significantly correlated with endothelial cell proliferation, underscoring its ability to detect newly formed, proliferating microvessels [24]. In melanoma, nestin was expressed in all T3 and T4 cases, while it was present in only half of the T2 or less severe cases. These findings suggest that the levels of nestin expression in melanoma are correlated with more advanced stages of the disease [26]. In another study, nestin was found to be expressed at varying levels in the majority of nodular melanomas (92%). Nestin expression was significantly correlated with increased tumor thickness, a high mitotic count, and the presence of ulceration and tumor necrosis [27].

Currently, nestin serves as a high-fidelity research tool for distinguishing between stable, mature vasculature and active neoangiogenesis. Unlike pan-endothelial markers (CD31/CD34), nestin-positive MVD allows for a dynamic assessment of the ‘angiogenic switch’ during the transition from actinic keratosis to SCC. Nestin is not a primary diagnostic marker for NMSC, as the diagnosis remains rooted in established histopathological criteria. However, it may serve as an auxiliary marker in challenging cases to highlight the microvascular reorganization characteristic of malignancy. While our data demonstrate a significant increase in nestin-positive MVD in SCC and BCC compared to AK, its role as an independent prognosticator of recurrence or metastasis remains speculative. While MVD has been linked to poor outcomes in other solid tumors, longitudinal studies are required to determine if nestin-specific MVD provides superior predictive value over standard tumor staging in skin cancer.

The findings of the present study warrant the examination of nestin-positive MVD across three distinct but interrelated clinical dimensions: diagnostic, prognostic, and therapeutic.

From a diagnostic perspective, nestin is not proposed as a replacement for established histopathological criteria in the diagnosis of non-melanoma skin cancer. However, nestin immunostaining may serve as an complementary marker in diagnostically challenging cases, particularly in small or superficial biopsies where the distinction between AK and early invasive SCC is equivocal on routine haematoxylin and eosin sections. The significantly elevated nestin-positive MVD observed in BCC and SCC compared to AK in the present study suggests that microvascular reorganization, as reflected by nestin expression, accompanies the transition from premalignant to malignant lesions. Nevertheless, this application requires prospective validation with defined sensitivity and specificity thresholds before any clinical implementation can be considered.

From a prognostic standpoint, MVD assessed with conventional markers such as CD31 and CD34 has been associated with adverse outcomes—including increased risk of recurrence, lymph node involvement, and reduced overall survival—across multiple solid tumor types, including colorectal, breast, and pancreatic carcinomas. In the present cohort, all SCC cases were non-metastatic at the time of diagnosis, precluding any direct analysis of the relationship between nestin-positive MVD and clinical outcomes. However, the observation that nestin-positive MVD was highest in SCC—the subtype carrying the greatest metastatic risk among non-melanoma skin cancers—is consistent with the hypothesis that elevated nestin-positive MVD may reflect a more aggressive angiogenic phenotype. Whether nestin-positive MVD independently predicts recurrence, depth of invasion beyond that already captured by Breslow-equivalent thickness, or metastatic potential in cutaneous SCC and BCC, remains to be established. Prospective longitudinal studies with adequate follow-up and clinical outcome data are required to determine whether nestin-positive MVD offers prognostic value incremental to current staging systems.

From a therapeutic perspective, the previous literature has explored the potential utility of nestin beyond passive biomarker status. Although nestin gene silencing has been demonstrated to inhibit endothelial cell proliferation in vitro, suggesting that nestin could theoretically serve as a functional target, it must be emphasized that no therapeutic application of nestin targeting has been evaluated in clinical trials. The translational relevance of these preclinical findings to cutaneous malignancies is currently unknown, and our study—which did not include mechanistic or interventional data—serves only to generate hypotheses in this regard.

Taken together, these considerations suggest that nestin-positive MVD may have a role across the diagnostic-to-therapeutic continuum in non-melanoma skin cancer; however, each of these potential applications requires dedicated prospective investigation before clinical translation can be contemplated.

This study has several limitations due to its retrospective, single-center design, which relied on the review of medical records and histopathology reports. All tissue samples were collected from a single dermatological clinic, which may introduce selection bias and limit the applicability of the results to diverse populations. The cross-sectional, retrospective nature of this study also precludes any longitudinal inference. Because tissue samples represent single time-point biopsies, it is not possible to track the dynamic evolution of MVD as individual lesions progress from AK to invasive carcinoma. Prospective longitudinal studies with sequential sampling would be required to establish a true temporal relationship between angiogenic marker expression and disease progression. Furthermore, the single-center setting limits the generalisability of the present findings to populations served by institutions with differing referral patterns, demographic profiles, Fitzpatrick skin phototypes, or sun-exposure histories.

Immunohistochemistry is a semi-quantitative method, and despite efforts to standardize it, the interpretation of staining intensity and distribution may vary among observers. Staining variability represents a further methodological constraint: differences in tissue fixation duration, antigen retrieval conditions, antibody lot, and chromogenic development time across cases may have introduced inconsistencies in signal intensity.

An additional limitation of this study is the absence of normal, sun-protected skin as a true negative control. AK, while representing a precursor lesion, is not biologically equivalent to normal skin, and itself exhibits alterations including inflammation and angiogenic remodeling. The inclusion of normal skin samples in future studies would provide a more complete baseline for understanding the progression of angiogenesis from normal skin through AK to invasive carcinomas. However, the focus of the present study was to compare nestin expression between premalignant and malignant lesions, for which AK serves as an appropriate and clinically relevant comparison group.

An additional methodological limitation of this study involves the strategy used for microvessel density (MVD) assessment. As dictated by the standardized criteria established by Weidner et al. [44], the three ‘hotspots’ were selected independently for each marker based on the highest concentration of nestin-, CD31-, or CD34-positive vessels. Because CD31 and CD34 detect both mature and newly formed vasculature, while nestin is primarily expressed in proliferating endothelial cells, the physiological hotspots for active neoangiogenesis may not strictly overlap spatially with hotspots of maximal overall vessel density.

Consequently, it is important to clarify that the strong positive correlations observed between nestin-positive MVD and conventional markers (CD31 and CD34) reflect broad, case-level trends in vascularity across the entire lesion. These data do not demonstrate direct spatial co-localization within identical micro-anatomical regions of interest (ROIs), as the hotspots analyzed for each marker often represented different geographical areas of the same tumor. While these correlations suggest that nestin expression increases in tandem with overall vessel density, they do not confirm that these markers are expressed on the same individual vessels. Future studies employing multiplex immunofluorescence or registration of serial sections could provide a more rigorous micro-anatomical comparison to determine the precise spatial overlap of these markers at the cellular level.

Therefore, our findings should be regarded as hypothesis-generating, and it is essential to conduct external validation in independent, prospectively recruited, multi-center cohorts before drawing any definitive conclusions about the utility of nestin as a complementary angiogenic marker.

## 5. Conclusions

In summary, our findings suggest that nestin may serve as a marker of angiogenesis in non-melanoma skin cancer. The mean density of nestin-positive vessels was significantly higher in BCC and SCC compared to AK. In adult tissues, nestin expression is characteristic of actively proliferating vascular endothelial cells, whereas mature vessels typically lack nestin expression. Accordingly, nestin has been proposed as a potential marker target for tumor-associated angiogenesis. However, this hypothesis requires confirmation in future prospective studies.

## Figures and Tables

**Table 1:** Characteristics of the study group.

	AK	BCC	SCC
No.	39	47	32
Age [years]:			
- Range	39–87	40–99	55–99
- Mean ± SD	70.4 ± 12.5	71.0 ± 12.9	78.6 ± 10.0
- Median	72	76	79
Sex			
- Female	28 (71.8%)	25 (53.2%)	11 (34.4%)
- Male	11 (28.2%)	22 (46.8%)	21 (65.6%)
Location			
- Sun-exposed skin	33 (84.6%)	41 (87.2%)	30 (93.8%)
- Covered skin	6 (15.4%)	6 (12.8%)	2 (6.2%)
Detailed location			
- Face	13 (33.3%)	22 (44.0%)	7 (21.9%)
- Nose	11 (28.2%)	10 (20.0%)	6 (18.8%)
- Lip	2 (5.1%)	1 (2.0%)	5 (15.6%)
- Ear	2 (5.1%)	6 (12.0%)	3 (9.4%)
- Head	1 (2.6%)	3 (6.0%)	2 (6.3%)
- Trunk	4 (10.3%)	6 (12.0%)	2 (6.3%)
- Upper limb	3 (7.7%)	1 (2.0%)	6 (18.8%)
- Lower limb	3 (7.7%)	1 (2.0%)	1 (3.1%)
Duration * [years]			
- Range	0.5–10	0.5–8	0.25–6
- Mean ± SD	1.4 ± 1.9	1.8 ± 1.75	1.5 ± 1.36
- Median	1	1	1
Infiltration depth [mm]			
- Range			1.2–18
- Mean ± SD			4.89 ± 4.2
- Median			3
Lesion diameter [mm]			
- Range	5–50		
- Mean ± SD	15.2 ± 9		
- Median	11		
Recurrent lesions		BCC recurrent: 4 (8.5%) BCC primary: 43 (91.5%)	
Multiple lesions		BCC multiple: 13 (27.7%) BCC single: 34 (72.3%)	

* Duration refers to the time from the patient’s first awareness of the lesion (based on clinical history) to the date of surgical excision and definitive histopathological diagnosis.

**Table 2:** Comparison of CD31-, CD34-, and nestin-positive microvessel density (MVD; vessels/mm^2^) in patients with AK.

Variable	Nestin	CD34	CD31
CD34	Pearson’s r: 0.69 *p*-value: <0.001	—	Pearson’s r: 0.41 *p*-value: 0.010
CD31	Pearson’s r: 0.46 *p*-value: 0.004	Pearson’s r: 0.41 *p*-value: 0.010	—

**Table 3:** Comparison of CD31-, CD34-, and nestin-positive microvessel density (MVD; vessels/mm^2^) in patients with BCC.

Variable	Nestin	CD34	CD31
CD34	Pearson’s r: 0.71 *p*-value: <0.001	—	Pearson’s r: 0.91 *p*-value: <0.001
CD31	Pearson’s r: 0.69 *p*-value: <0.001	Pearson’s r: 0.91 *p*-value: <0.001	—

**Table 4:** Comparison of CD31-, CD34-, and nestin-positive microvessel density (MVD; vessels/mm^2^) in patients with SCC.

Variable	Nestin	CD34	CD31
CD34	Pearson’s r: 0.82 *p*-value: <0.001	—	Pearson’s r: 0.86 *p*-value: <0.001
CD31	Pearson’s r: 0.78 *p*-value: <0.001	Pearson’s r: 0.86 *p*-value: <0.001	—

**Table 5:** Kruskal–Wallis test comparing nestin-positive microvessel density (MVD; vessels/mm^2^) in actinic keratosis (AK), basal cell carcinoma (BCC), and squamous cell carcinoma (SCC).

Marker	Tumor Type	Mean Rank	M	Me	SD	95% CI	H(2)	*p*-Value	η^2^
Nestin	AK	29.13	10.15	9.66	5.45	[8.39–11.92]	48.61	<0.001	0.41
	BCC	69.31	18.09	17.67	4.73	[16.70–19.48]			
	SCC	82.11	20.99	21.33	6.60	[18.61–23.37]			
CD31	AK	33.58	13.86	12.33	6.95	[11.61–16.11]	38.00	<0.001	0.310
	BCC	66.46	20.91	21.67	5.94	[19.16–22.65]			
	SCC	82.03	24.87	24.00	7.32	[22.27–27.46]			
CD34	AK	31.35	13.68	12.00	6.61	[11.53–15.82]	41.77	<0.001	0.346
	BCC	68.61	20.99	21.33	5.98	[19.23–22.74]			
	SCC	80.44	23.67	23.67	6.86	[21.39–26.34]			

M, mean; Me, median; SD, standard deviation; H(2), 95% CI, 95% confidence interval; Kruskal–Wallis H statistic (degrees of freedom = 2); η^2^, eta-squared effect size. All MVD values are expressed as the mean number of positively stained vessels per square millimeter (vessels/mm^2^), counted in three hot spot areas of 0.95 mm^2^ each at 200× magnification.

**Table 6:** *Post hoc* Dunn–Bonferroni comparison of nestin-positive microvessel density (MVD; vessels/mm^2^) in AK, BCC, and SCC.

Marker	Compared Groups (Group 1–Group 2)	Mean MVD in Group 1	Mean MVD in Group 2	Test Statistic	Standard Error	Standardized Test Statistic	Rank-Biserial r	Cohen’s d	*p*
	AK–BCC	10.15	18.09	41.47	7.39	5.60	0.745	−1.565	<0.001
Nestin	AK–SCC	10.15	20.99	54.05	8.13	6.64	0.804	−1.807	<0.001
	BCC–SCC	18.09	20.99	12.57	7.77	1.62	0.295	−0.521	0.077
	AK–BCC	13.86	20.91	32.88	7.47	4.40	0.609	−1.098	<0.001
CD31	AK–SCC	13.86	24.87	48.45	8.16	5.94	0.733	−1.546	<0.001
	BCC–SCC	20.91	24.87	15.57	7.83	1.99	0.329	−0.605	0.141
	AK–BCC	13.68	20.99	37.26	7.41	5.03	0.668	−1.166	<0.001
CD34	AK–SCC	13.68	23.67	49.09	8.16	6.02	0.779	−1.516	<0.001
	BCC–SCC	20.99	23.67	11.83	7.84	1.51	0.245	−0.454	0.394

AK, actinic keratosis; BCC, basal cell carcinoma; SCC, squamous cell carcinoma; MVD, microvessel density. Mean MVD values are expressed as the mean number of positively stained vessels per square millimeter (vessels/mm^2^). Effect sizes are reported as rank-biserial r for all markers; Cohen’s d is additionally reported for all comparisons. All *p*-values are Bonferroni-corrected following *post hoc* Dunn–Bonferroni correction.

## Data Availability

The data presented in this study are available on request from the corresponding author due to privacy and legal restrictions.
